# The peripheral differentiation of human natural killer T cells

**DOI:** 10.1111/imcb.12248

**Published:** 2019-04-08

**Authors:** Jie Liu, Brenna J Hill, Sam Darko, Kaimei Song, Máire F Quigley, Tedi E Asher, Yohei Morita, Hui Y Greenaway, Vanessa Venturi, Daniel C Douek, Miles P Davenport, David A Price, Mario Roederer

**Affiliations:** ^1^ Laboratory of Infectious Diseases and Vaccines State Key Laboratory of Biotherapy West China Hospital Sichuan University Chengdu 610041 PR China; ^2^ ImmunoTechnology Section Vaccine Research Center National Institute of Allergy and Infectious Diseases National Institutes of Health Bethesda MD 20892 USA; ^3^ Human Immunology Section Vaccine Research Center National Institute of Allergy and Infectious Diseases National Institutes of Health Bethesda MD 20892 USA; ^4^ Division of Infection and Immunity Cardiff University School of Medicine Heath Park Cardiff CF14 4XN UK; ^5^ Leibniz Institute on Aging Fritz Lipmann Institute 07745 Jena Germany; ^6^ Infection Analytics Program Kirby Institute for Infection and Immunity University of New South Wales Sydney Sydney NSW 2052 Australia

**Keywords:** NKT cell, T‐cell differentiation, TCR, TREC

## Abstract

The peripheral maturation of human CD1d‐restricted natural killer T (NKT) cells has not been well described. In this study, we identified four major subsets of NKT cells in adults, distinguished by the expression of CD4, CD8 and CCR5. Phenotypic analysis suggested a hierarchical pattern of differentiation, whereby immature CD4^+^ CD8^−^ CCR5^−^ cells progressed to an intermediate CD4^+^ CD8^−^ CCR5^+^ stage, which remained less differentiated than the CD4^−^ CD8^−^ and CD4^−^ CD8^+^ subsets, both of which expressed CCR5. This interpretation was supported by functional data, including clonogenic potential and cytokine secretion profiles, as well as T‐cell receptor (TCR) excision circle analysis. Moreover, conventional and high‐throughput sequencing of the corresponding TCR repertoires demonstrated significant clonotypic overlap within individuals, especially between the more differentiated CD4^−^ CD8^−^ and CD4^−^ CD8^+^ subsets. Collectively, these results mapped a linear differentiation pathway across the post‐thymic landscape of human CD1d‐restricted NKT cells.

## Introduction

Classical natural killer T (NKT) cells express a highly biased T‐cell receptor (TCR) repertoire and serve as a bridge between the innate and adaptive immune systems.^1^, ^2^ The semi‐invariant human NKT cell TCR comprises a fixed *TRAV10/TRAJ18* gene‐encoded α‐chain with a canonical CDR3α loop^3^ and a restricted *TRBV25* gene‐encoded β‐chain, which together enable the recognition of glycolipids presented in the context of CD1d.^4^ NKT cells deploy a range of effector functions in response to antigen encounter and contribute in various ways to the immune processes that mediate pathogen control, tumor surveillance, allergic phenomena and autoimmune disorders.^5^

Although initially considered to be homogeneous, later studies revealed considerable phenotypic and functional diversity within the peripheral NKT cell compartment. Two subsets, CD4^+^CD8^−^ and CD4^−^CD8^−^, have been described in mice, and a third subset, CD4^−^CD8^+^, has been described in humans.^6^ These patterns of coreceptor use segregate with functionally distinct effector programs.^7^, ^8^ The development of murine NKT cells is thought to comprise four stages, based on the expression of CD24, CD44 and NK1.1.^9^, ^10^ Further nuances are suggested by the existence of mature PLZF^high^Tbet^low^RORγt^low^ interleukin (IL)‐4‐producing and PLZF^high^Tbet^low^RORγt^high^ IL‐17‐producing subsets in the thymus that resemble NK1.1^−^ NKT cells.^11^, ^12^ It is also likely that peripheral CD4^+^ and CD4^−^ NKT cells in mice represent distinct lineages that emigrate independently from the thymus.^13^ However, the extent to which human NKT cells follow an equivalent differentiation pathway remains unclear, despite close parallels in the TCR‐mediated antigen recognition process and the highly conserved nature of CD1d.

In this study, we combined phenotypic, functional and molecular techniques to characterize the post‐thymic differentiation of human NKT cells. Our data supported the notion of a single lineage compartment and outlined a maturation pathway compatible with the reported heterogeneity among circulating subsets of CD1d‐restricted NKT cells.

## Results

### Identification of NKT cells

**Figure 1:**
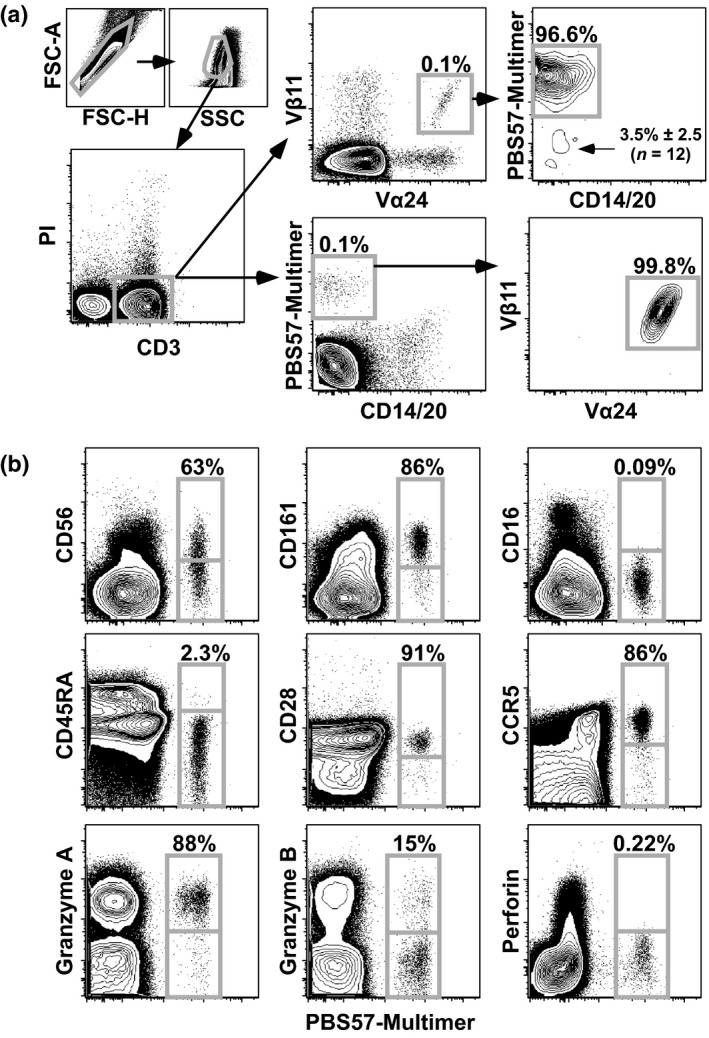
Identification and phenotype of human NKT cells. **(a)** Viable CD3^+^ cells (left) were assessed for TRAV10 (Vα24) and TRBV25 (Vβ11) expression (top right) or PBS57‐hCD1d multimer binding (bottom right). Data are representative of three independent experiments (*n* = 5–12 subjects). PI, propidium iodide. **(b)** Expression of the indicated phenotypic markers is shown for NKT (PBS57‐hCD1d^+^) and conventional T cells (PBS57‐hCD1d^−^). Plots are gated on viable CD3^+^ cells. Data are representative of five independent experiments (*n* = 12 subjects). Summary statistics are provided in Table 1.

Historically, NKT cells were identified by the expression of TRAV10/TRBV25 heterodimeric TCRαβ complexes.^14^, ^15^ More recently, multimers of human CD1d (hCD1d) incorporating one of two different glycolipids (αGalCer or PBS57) have been used to detect NKT cells on the basis of antigen specificity.^16^, ^17^, ^18^ As shown in Figure 1a, a vast majority of CD3^+^ PBS57‐hCD1d multimer‐binding cells expressed the invariant TCR. Among total peripheral blood mononuclear cells (PBMCs), only 0.08% ± 0.06 (*n* = 12) of multimer‐binding cells lacked CD3, thereby demonstrating both the exquisite specificity and selectivity of this reagent. In contrast, dual staining for TRAV10 (Vα24) and TRBV25 (Vβ11) revealed a small population of non‐NKT cells (3.5% ± 2.5; *n* = 12). We therefore used the PBS57‐hCD1d multimer to characterize NKT cells.

### Phenotypic analysis of NKT cells

To define the properties of PBS57‐hCD1d multimer‐binding cells, we initially measured several proteins typically expressed by T and/or NK cells (Figure 1b and Table 1). The NK cell markers CD56 and CD161 were found at much higher frequencies on NKT cells compared with conventional T cells. In contrast, NKT cells almost completely lacked CD16 and infrequently expressed the NK recognition molecules NKB1 and NKAT2. These results largely concurred with a previous report that used the monoclonal antibody 6B11 to identify NKT cells.^19^ However, there were two notable exceptions. First, we found that nearly all NKT cells expressed CD95, consistent with a predominant memory phenotype.^20^ Second, we found that a higher fraction of NKT cells expressed CD56. These differences likely reflected greater sensitivity in our flow cytometry panel for these dimly expressed proteins.

**Table 1:** NKT cell expression of surface markers

Marker	% of total T cells	% of NKT cells
NK‐associated markers		
CD16	2.4 ± 0.6	0.1 ± 0.2
CD56	5.0 ± 2.8	62.8 ± 29.0
CD161	10.4 ± 2.6	86.1 ± 14.6
NKB1	1.7 ± 0.6	4.6 ± 3.3
NKAT2	2.4 ± 2.1	0.2 ± 0.1
Differentiation‐associated markers		
CD7	92.0 ± 2.6	96.8 ± 2.3
CD11a^high^	40.5 ± 12.1	94.3 ± 2.9
CD27	86.9 ± 5.3	86.1 ± 5.2
CD28	71.1 ± 5.6	90.5 ± 5.9
CD45RA	38.8 ± 6.6	2.3 ± 1.2
CD57	5.1 ± 3.1	1.3 ± 0.7
CD62L	76.0 ± 2.8	7.5 ± 1.4
CD95	57.3 ± 7.3	92.0 ± 3.2
CD127	46.7 ± 1.2	76.2 ± 6.1
CCR5	15.3 ± 6.0	86.3 ± 7.6
Cytolytic enzymes		
Granzyme A	17.0 ± 10.36	88.2 ± 2.8
Granzyme B	6.1 ± 4.5	15.0 ± 4.6
Perforin	2.6 ± 2.4	0.2 ± 0.4
Lineage markers		
CD4	68.7 ± 10.1	15.2 ± 9.0
CD8	22.1 ± 8.7	19.5 ± 6.6

Percentages are given as mean ± one standard deviation for 5–12 subjects.

Most NKT cells expressed CCR5 but lacked CD45RA and CD62L, a phenotype reminiscent of effector T cells. However, similarly high frequencies of NKT cells expressed CD27, CD28 and CD127, markers typically associated with central memory T cells. The expression of cytolytic enzymes was also consistent with a central memory phenotype, in that most NKT cells expressed granzyme A, whereas only a few expressed granzyme B, and virtually none expressed perforin. NKT cells therefore occupied a unique phenotypic niche most akin to central memory T cells.

### Subsets of NKT cells

**Figure 2:**
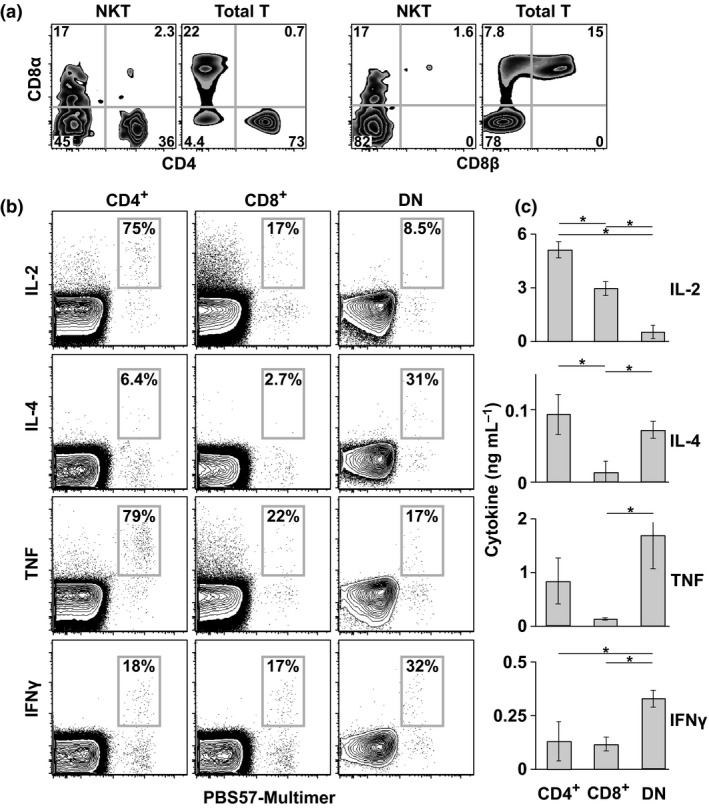
Subsets of NKT cells. **(a)** Expression of CD4, CD8α and CD8β is shown for NKT (PBS57‐hCD1d^+^) and conventional T cells (PBS57‐hCD1d^−^) from a representative individual. Plots are gated on viable CD3^+^ cells. Quadrant numbers indicate percentage values. The percentages are averaged across three independent experiments (*n* = 7 subjects). **(b)** Expression of the indicated cytokines is shown for CD4^+^, DN and CD8^+^ NKT cells from a representative individual. The percentages are averaged across two independent experiments (*n* = 5 subjects). **(c)** CD4^+^, DN and CD8^+^ NKT cells (*n* = 10 000 per subset) were stimulated with the PBS57‐hCD1d multimer. The amount of each cytokine released into the supernatant was measured using cytokine bead array. Results are shown as the mean ± one standard deviation from two independent experiments (*n* = 3–5 subjects). *P < 0.05 (Kruskal–Wallis test with Steel–Dwass correction).

Analysis of the T‐cell lineage markers CD4 and CD8 confirmed that NKT cells could be divided into three subsets: CD4^+^CD8^−^ (CD4^+^), CD4^−^CD8^−^ (DN) and CD4^−^CD8^+^ (CD8^+^) (Figure 2a). Unlike conventional CD8αβ^+^ T cells, but allied to NK and γδ T cells, NKT cells expressed CD8 exclusively as the homodimer CD8αα.

### Functional analysis of NKT cells

To determine the functional characteristics of NKT cells, we stimulated PBMCs with αGalCer, anti‐CD3 or PBS57‐hCD1d in the presence of brefeldin A and measured the expression of intracellular cytokines, namely IL‐2, IL‐4, TNF and IFNγ. The PBS57‐hCD1d multimer emerged as the most potent stimulant (data not shown). In line with the division‐linked heterogeneity of memory T cells, functional differences were evident among phenotypically defined subsets of NKT cells (Figure 2b). In particular, we found that CD4^+^ and DN NKT cells were significant sources of IL‐4, as reported previously.^7^, ^8^, ^19^, ^21^, ^22^, ^23^ Large fractions of CD4^+^ NKT cells also expressed high levels of IL‐2 and TNF compared with CD4^−^ NKT cells. In contrast, CD4^−^ NKT cells more frequently expressed IFNγ. These patterns of cytokine secretion suggested that CD4^+^ NKT cells were less differentiated than CD4^−^ NKT cells.

In further experiments, we sorted NKT cell subsets to purity and quantified extracellular cytokine secretion in response to stimulation with the PBS57‐hCD1d multimer (Figure 2c). These measurements were largely concordant with the intracellular cytokine profiles, barring a few minor discrepancies likely attributable to methodological differences. One notable exception was the finding that DN NKT cells produced high levels of TNF, which suggested greater output on a per cell basis relative to CD4^+^ or CD8^+^ NKT cells.

### Differentiation of NKT cells

**Figure 3:**
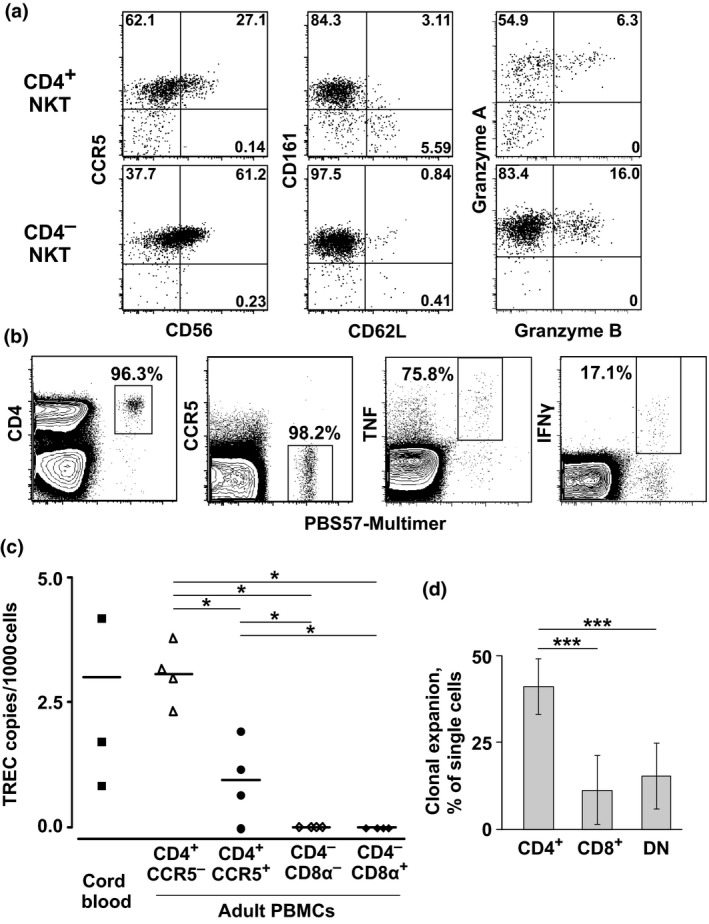
Differentiation of NKT cells. **(a)** Expression of the indicated phenotypic markers is shown for CD4^+^ (top) and CD4^−^ NKT cells (bottom) from a representative individual. Quadrant numbers indicate percentage values. The percentages are averaged across four independent experiments (*n* = 12 subjects). **(b)** Expression of the indicated phenotypic markers (left panels) and cytokines produced in response to stimulation with the PBS57‐hCD1d multimer (right panels) is shown for cord blood NKT (PBS57‐hCD1d^+^) and conventional T cells (PBS57‐hCD1d^−^) from a representative individual. The percentages are averaged across four independent experiments (*n* = 5 subjects). **(c)** TREC levels were quantified in sort‐purified NKT cell subsets. Horizontal bars indicate mean values. Each data point represents one individual. *P < 0.05 (Kruskal–Wallis test with uncorrected pairwise comparisons for adult PBMCs). **(d)** Single CD4^+^, DN and CD8^+^ NKT cells were sorted and cloned. The fraction of cells that expanded *in vitro* is shown as the mean ± one standard deviation from three independent experiments (*n* = 3 or 4 subjects). ***P < 0.005 (Kruskal–Wallis test with Steel–Dwass correction).

On the basis of these functional traits, we hypothesized that CD4^+^ NKT cells were less differentiated than CD4^−^ NKT cells. This inference was substantiated by comparing markers of differentiation across NKT cell subsets (Table 2). Fewer CD4^+^ NKT cells expressed CD11a, CCR5, granzymes A and B, and NK‐associated markers compared with CD4^−^ NKT cells. In contrast, CD4^+^ NKT cells more frequently expressed CD62L. Careful analysis further revealed distinct subsets of CD4^+^ NKT cells. The smaller population, representing about 5–10% of CD4^+^ NKT cells, expressed CD62L but lacked CCR5, CD161 and granzyme A (Figure 3a and Supplementary figure 1). This phenotype was reminiscent of naïve T cells, with the exception that NKT cells rarely expressed CD45RA. The CCR5^−^ subset therefore appeared to be less differentiated than the CCR5^+^ subset within the CD4^+^ NKT cell compartment.

**Table 2:** Phenotypic comparison of T and NKT cell subsets

Marker	T cells		NKT cells		
	% of CD4^+^	% of CD8^+^	% of CD4^+^	% of CD8^+^	% of DN
NK‐associated markers					
CD56	1.1 ± 1.2	7.8 ± 6.1	42.1 ± 16.3	75.4 ± 18.2	79.1 ± 12.7
CD161	8.9 ± 5.0	9.9 ± 4.8	62.1 ± 25.4	88.7 ± 10.6	92.1 ± 8.2
NKB1	0.2 ± 0.1	2.1 ± 0.8	0.6 ± 0.5	9.4 ± 2.5	18.6 ± 2.1
Differentiation‐associated markers					
CD11a^high^	38.4 ± 6.4	40.6 ± 12.0	78.0 ± 13.9	96.3 ± 2.4	93.6 ± 4.9
CD62L	82.5 ± 2.5	60.8 ± 16.6	22.4 ± 15.1	4.9 ± 2.4	4.3 ± 1.1
CD95	54.3 ± 5.7	40.8 ± 16.8	89.6 ± 6.8	91.6 ± 2.1	93.4 ± 2.7
CD127	42.1 ± 7.5	45.9 ± 4.8	70.3 ± 12.6	77.3 ± 10.6	77.2 ± 13.4
CCR5	9.1 ± 4.2	28.1 ± 13.1	63.3 ± 20.1	85.2 ± 17.2	90.0 ± 12.1
Cytolytic enzymes					
Granzyme A	3.7 ± 1.3	41.4 ± 14.5	56.1 ± 21.2	95.1 ± 9.7	96.8 ± 2.3
Granzyme B	0.3 ± 0.2	11.0 ± 11.7	4.3 ± 7.4	25.3 ± 10.1	8.45 ± 4.1

Percentages are given as mean ± one standard deviation for 5–7 subjects.

To confirm these findings, we undertook several independent lines of experimentation. First, we characterized NKT cells in cord blood (Figure 3b). These progenitors were found to be uniformly CD4^+^CCR5^−^ (and still CD45RA^−^), with a functional profile similar to that of adult CD4^+^ NKT cells (high expression of TNF and relatively low expression of IFNγ). Second, we quantified T‐cell receptor excision circles (TRECs) in NKT cell subsets. TREC levels decline geometrically with the number of divisions since rearrangement of the TCR.^24^ As shown in Figure 3c, the highest TREC levels were detected in adult and cord blood CD4^+^CCR5^−^ NKT cells. This subset therefore emerged during the initial stages of development. Progressively lower TREC levels were present in CD4^+^CCR5^+^ and CD4^−^ NKT cells. Third, we cloned phenotypically defined NKT cells. In line with previous studies of *in vitro* proliferation,^25^, ^26^ we found that CD4^+^ NKT cells were significantly more amenable to clonogenic expansion compared with CD4^−^ NKT cells (Figure 3d). It was also notable that CD4 persisted on the surface of all expanded CD4^+^ NKT cell clones (data not shown). This finding suggested that any transition to the CD4^−^ state was either rare or contingent on additional stimuli, such as further proliferation or an unknown *in vivo* signal. Similarly, both DN and CD8^+^ NKT cells largely retained their phenotypes in culture, although there was some plasticity in the expression of CD8α. Most CD8^+^ clones became heterogeneous in this respect, and the occasional DN clone acquired CD8α.

### Clonotypic analysis of NKT cell subsets

To probe these lineage relationships in more detail, we performed an unbiased molecular analysis of all expressed *TR* gene products in sort‐purified (> 98%) subsets of NKT cells. The flow cytometric sorting strategy is shown in Supplementary figure 2.

**Figure 4:**
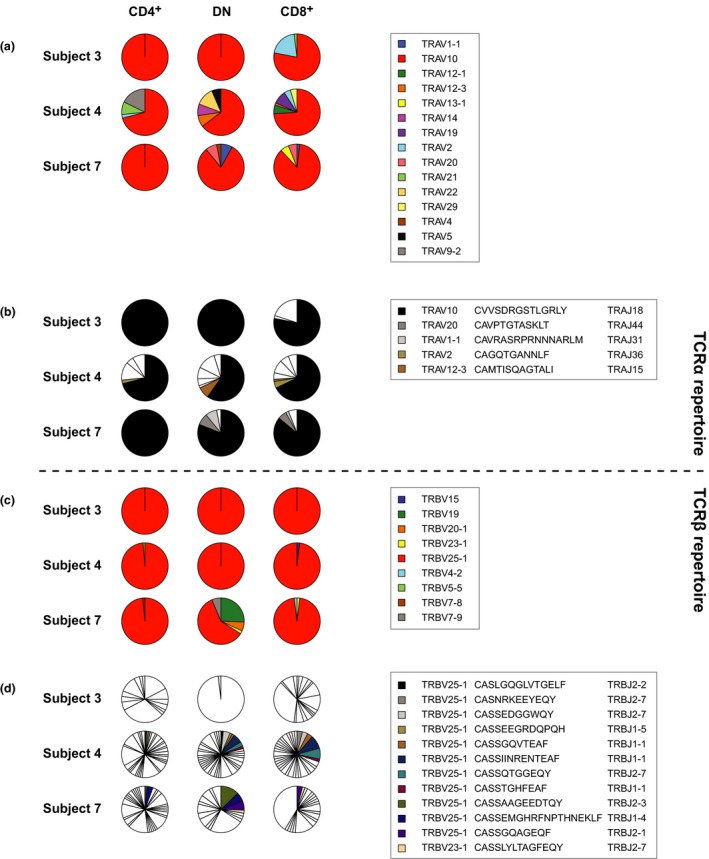
Conventional analysis of TCR use in NKT cell subsets. CD4^+^, DN and CD8^+^ NKT cells were sort‐purified from healthy subjects (*n* = 3). A conventional sequencing approach was used to profile the corresponding TCRα and TCRβ repertoires. The fraction of each repertoire expressing a particular *TRAV* **(a)** or *TRBV* gene **(c)** is shown together with the fraction of each repertoire expressing a specific TCRα **(b)** or TCRβ sequence **(d)**. The invariant TCRα sequence TRAV10/CVVSDRGSTLGRLY/TRAJ18 is shown in black.

In a cross‐sectional analysis of three healthy subjects, we found that the canonical TRAV10/CVVSDRGSTLGRLY/TRAJ18 sequence^14^, ^15^, ^27^ was ubiquitous and highly conserved at the nucleotide level among CD4^+^, DN and CD8^+^ NKT cells (Figure 4a, b). Some additional TCRα sequences were detected, especially in subject 4, presumably reflecting a lack of allelic exclusion. In line with previous reports,^28^ the corresponding TCRβ sequences were substantially more diverse and predominantly TRBV25‐1^+^ (Figure 4c, d). Importantly, we found nucleotide‐identical TCRβ clonotypes within all three phenotypically defined subsets from subject 4 and subject 7, thereby providing direct evidence that CD4^+^, DN and CD8^+^ NKT cells were related by ancestry and/or interconversion. Our data were significant in this context. Assuming a null hypothesis that each subset arose independently, equivalent sharing of TCRβ sequences would have been expected among CD4^+^, DN and CD8^+^ NKT cells both within and between subjects. This scenario was rejected (*P* < 10^−4^; Fisher's exact test).

**Figure 5:**
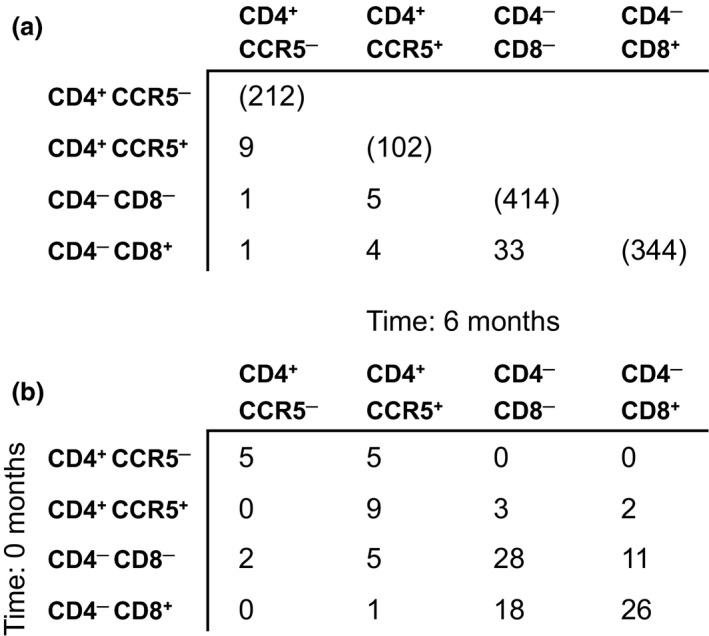
High‐throughput analysis of TCR use in NKT cell subsets. CD4^+^ CCR5^−^, CD4^+^ CCR5^+^, DN and CD8^+^ NKT cells were sort‐purified from healthy subjects (*n* = 3). A high‐throughput sequencing approach was used to profile the corresponding TCRβ repertoires. The experiment was repeated 6 months later. **(a)** The number of unique TCRβ sequences identified for each NKT cell subset is listed in parentheses. Numbers below the diagonal indicate how many of these unique TCRβ sequences were shared across NKT cell subsets from the same individual at a single time point. **(b)** The number of shared unique TCRβ sequences is shown for each combination of NKT cell subsets within an individual at the indicated time points.

To confirm and extend these findings, we used high‐throughput sequencing to analyze TCRβ sharing across NKT cell subsets in three additional healthy subjects. In each case, two samples of peripheral blood were taken 6 months apart, thereby enabling an assessment of clonotype distribution over time. Moreover, the CD4^+^ NKT cell subset was further sort‐purified on the basis of CCR5 expression. In line with our conventional sequencing analysis, we found shared clonotypes at individual time points across all four phenotypically defined subsets of NKT cells (Figure 5a). The most extensive overlap was detected between DN and CD8^+^ NKT cells. This pattern was recapitulated in the longitudinal dataset, suggesting a high degree of fluidity between these phenotypes (Figure 5b). Relatively few TCRβ sequences were shared over time, however, especially between the CD4^+^ and CD4^−^ NKT cell subsets. A few CD4^+^CCR5^+^ clonotypes nonetheless appeared to seed the DN and CD8^+^ populations. In contrast, no transitions were observed back to the CD4^+^CCR5^−^ state. Collectively, these data provided evidence for linear differentiation within a highly dynamic system.

## Discussion

Human NKT cells were classified previously into three broad subsets, defined on the basis of coreceptor expression (CD4^+^, DN and CD8^+^). Earlier work also suggested that NKT precursors expressed CD4 but not CD161. This view was upheld by the observation that CD4^+^ NKT cells predominated both in cord blood and in the thymus.^29^, ^30^, ^31^ Moreover, the expression of CD161 was shown to increase with age in young humans,^30^ consistent with a progressive maturation of the NKT cell compartment. In line with previous studies,^8^, ^32^, ^33^ we found that CD4^+^ NKT cells could be subdivided according to the expression of CCR5. Our collated phenotypic, functional and molecular data further indicated that these subsets represented distinct stages of maturation within the post‐thymic NKT cell pool.

CD4^+^CCR5^−^ NKT cells likely emerged first in the periphery. These cells expressed many markers commonly associated with a naïve phenotype among conventional T cells (CD27, CD28, CD62L, CD127 and low CD11a) and lacked cytolytic enzymes (granzymes A and B, and perforin). They also harbored TREC levels comparable to those found in cord blood NKT cells, which shared all of the same phenotypes. This finding suggested that CD4^+^CCR5^−^ NKT cells were produced continuously throughout adulthood. However, these cells were clearly not naïve in the classical sense, because they did not express CD45RA and could be triggered in some instances to produce IFNγ.

CD4^+^CCR5^+^ NKT cells appeared to be more differentiated. They had lower TREC levels, consistent with an average of two additional divisions from the CCR5^−^ stage, and readily produced effector cytokines upon restimulation. During *in vitro* clonal expansion, however, these cells largely maintained their phenotype, suggesting that further transition was either rare or dependent on other stimuli, such as additional division cycles or a signal that was not recapitulated in culture. In line with this interpretation, distinct tissue‐specific microenvironments were shown previously to influence both the differentiation^34^ and function^35^, ^36^, ^37^ of murine NKT cells.

CD4^−^ NKT cells were either CD8^−^ or CD8αα^+^ (none expressed CD8β). Our data suggested that the expression of CD8αα on CD4^−^ NKT cells was reversible, potentially reflecting the promiscuity of the α‐chain promoter upon T‐cell stimulation. On this basis, it was conceivable that CD8^+^ NKT cells arose as a consequence of *in vivo* activation rather than programmed differentiation, with functional differences mirroring the chronology of antigen encounter on a cell‐by‐cell basis. This notion was supported by the clonotypic relationship between DN and CD8^+^ NKT cells. In comparison with the CD4^+^ subsets, CD4^−^ NKT cells displayed lower clonogenic potential and greater functionality, particularly with regard to the expression of cytolytic enzymes. These features indicated a more differentiated phenotype, substantiated by the relative absence of TRECs.

DN and CD8^+^ NKT cells were rarely terminally differentiated in the classical sense, instead maintaining a “central memory‐like” phenotype in relation to conventional T cells (CD27^+^CD28^+^CD127^+^). However, the latter seldom expressed these molecules alongside CCR5. This key difference potentially reflected contrasting selection processes in the thymus and/or divergent programming at the transcriptional level.^13^ In practical terms, the expression of CCR5 indicated that differentiated NKT cells were poised to mobilize rapidly and participate in early immune responses triggered by the release of CC‐chemokines.^38^

**Figure 6:**
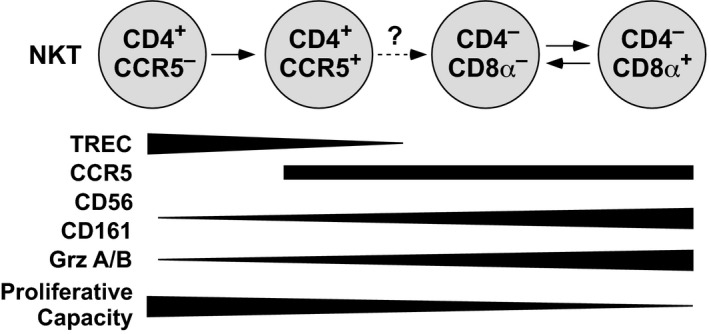
The peripheral ontogeny of human NKT cells. Based on the phenotypic, functional and molecular data presented in this study, we propose a linear differentiation model that describes the peripheral ontogeny of human NKT cells. The depicted lineage relationships are consistent with previous reports, as reviewed in detail elsewhere.^13^

Collectively, these results provided evidence for a linear differentiation pathway, as depicted in Figure 6. The most likely alternative scenario was that CD4^+^ and CD4^−^ NKT cells arose separately as distinct lineages in the thymus. However, this latter model failed to explain why CD4^−^ NKT cells divided and differentiated in the periphery to a much greater extent than CD4^+^ NKT cells. It was also difficult to reconcile independent recombination events with the contemporaneous occurrence and longitudinal sharing of identical TCRβ clonotypes between the CD4^−^ and CD4^+^ subsets. Accordingly, our findings were most consistent with a single lineage compartment and offered a working hypothesis to clarify the peripheral ontogeny of human NKT cells.

## Methods

### Subjects

Adult blood samples were obtained from male and female healthy volunteers aged 23–45 years, recruited via the Blood Bank at the National Institutes of Health (Bethesda, MD). Cord blood samples were obtained from healthy newborn infants delivered full‐term at the Shady Grove Hospital Center (Gaithersburg, MD). The use of human samples for this work was approved by the Institutional Review Board of the National Institute of Allergy and Infectious Diseases at the National Institutes of Health.

### Culture medium, antibodies and dyes

PBMCs were isolated via standard density gradient centrifugation and maintained in RPMI 1640 medium supplemented with 10% fetal bovine serum, 2 mm l‐glutamine, 100 μg mL^−1^ streptomycin and 100 U mL^−1^ penicillin (all from Life Technologies, San Diego, CA, USA). Freshly prepared cells for analysis in the absence of *in vitro* stimulation were either used directly in flow cytometric experiments or placed in culture overnight for concurrent analysis with stimulated cells. All antibody reagents, either purified or pre‐conjugated, were obtained from BD Biosciences (San Diego, CA, USA), except (1) anti‐TRAV10 (Vα24), anti‐TRBV25 (Vβ11) and anti‐CD127 (Beckman Coulter, Brea, CA, USA); (2) anti‐granzyme B (Caltag, Burlingame, CA, USA); and (iii) anti‐CCR7 (R&D Systems, Minneapolis, MN, USA). Multimeric complexes of PBS57‐hCD1d, a ligand analog of αGalCer‐CD1d, were obtained from the National Institutes of Health Tetramer Facility.

### Flow cytometric analysis

Standard procedures were used to identify cell surface markers and intracellular molecules. Processed samples were collected using a modified LSRII flow cytometer (BD Biosciences) configured as described previously.^39^ Data were analyzed with FlowJo software version 8.1.1 (FlowJo, LLC, Ashland, OR, USA).

### Clonal expansion of NKT cells

Single cells from CD4^+^, DN and CD8^+^ NKT cell subsets were sorted into individual wells of a 96‐well plate using a modified FACSAria flow cytometer (BD Biosciences). Allogeneic PBMCs from a healthy subject were loaded with αGalCer (100 ng mL^−1^; Enzo Life Sciences, Farmingdale, NY, USA) and irradiated (4000 rad) for use as antigen‐presenting cells. The cultures were supplemented with IL‐2 (100 U mL^−1^) and IL‐7 (10 pg mL^−1^) and replenished with fresh medium every 3 days. Active clones were transferred to 24‐well plates after 9 days and harvested for phenotypic analysis by flow cytometry after further expansion.

### Intracellular cytokine assay

PBMCs were suspended in culture medium with PBS57‐hCD1d (0.2 μg mL^−1^), anti‐CD28 (1 μg mL^−1^) and anti‐CD49d (1 μg mL^−1^) for 12 h at 37°C. Brefeldin A was added at a concentration of 1 μg mL^−1^ for the final 8 h. The cells were then washed, stained for lineage/differentiation markers, washed again, fixed/permeabilized using Cytofix/Cytoperm (BD Biosciences) and stained for intracellular cytokines. After a final wash step, the cells were fixed and stored at 4°C pending analysis.

### Cytokine bead array

Cytokine production was assessed via cytometric bead array using a Human Th1/Th2 Cytokine Kit (BD Biosciences). Briefly, PBMCs were stained with the PBS57‐hCD1d multimer and directly conjugated antibodies to identify NKT cell subsets, then sorted in bulk using a modified FACSAria flow cytometer (BD Biosciences). Sorted cells were cultured in a 96‐well plate at 10 000 cells/50 μL per well in the presence of anti‐CD28 (1 μg mL^−1^) and anti‐CD49d (1 μg mL^−1^) with or without PBS57‐hCD1d (0.2 μg mL^−1^) for 18 h at 37°C. Supernatants were recovered after incubation and assessed for cytokine production by flow cytometry using capture beads.

### TREC analysis

TREC frequencies were evaluated via quantitative real‐time PCR (qRT‐PCR) as described previously.^24^ Briefly, NKT cell subsets were sorted in bulk as described above and lysed with proteinase K. The solubilized DNA was then used directly as the amplification template. qRT‐PCR was performed in duplicate tubes against a standard curve generated from serial dilutions of TREC plasmid DNA.

### Conventional TCR sequencing and data analysis

Viable NKT cells (1000–10 000 per subset) were sorted by flow cytometry as described above to > 98% purity. Molecular analysis of expressed *TR* gene rearrangements was then performed using a template‐switch anchored RT‐PCR according to reported protocols.^40^, ^41^, ^42^ A minimum of 50 functional sequences was analyzed for each sorted NKT cell population. Each TCR sequence was aligned against all reference *TRAV* and *TRAJ* or *TRBV*,* TRBD* and *TRBJ* gene sequences from the IMGT database.^43^ The *V* genes were aligned first, followed by the *J* genes and, for *TRB* sequences, the *D* genes. The *V* and *J* genes were assigned as the longest, highest percentage identity match with the TCR sequence. Clonotype identity across subsets within individuals was defined by precise matching of nucleotide sequences.

### High‐throughput TCR sequencing and data analysis

Libraries were constructed as described previously.^44^ Briefly, mRNA was extracted from sorted cells using a μMACS mRNA Isolation Kit (Miltenyi Biotec, Sunnyvale, CA, USA). A template‐switch anchored RT‐PCR was then performed using a modified version of the SMARTer RACE cDNA Amplification Kit (Clontech, Mountain View, CA, USA). After cDNA synthesis and purification, TCRβ templates were amplified using a KAPA Real Time Library Amplification Kit (Kapa Biosystems, Wilmington, MA, USA), with a *TRBC* gene‐specific 3′ primer (5′‐GCTTCTGATGGCTCAAACACAGCGACCT‐3′) and a standard 5′ primer (5 Primer II A; Clontech). Amplified samples were separated using an E‐Gel Size Select 2% Gel System (Life Technologies). Illumina adaptors were added via a second PCR using the KAPA Real Time Library Amplification Kit (Kapa Biosystems), incorporating 20 μL of each library with one 3′ primer (5′‐CAAGCAGAAGACGGCATACGAGATTGCTTCTGATGGCTCAAACACAGCGACCT‐3′) and two 5′ primers (PE1 FCB ILL 1_2 and the corresponding PE1 ILL barcode 2_2 primer). PCR products were cleaned using an AMPure XP Kit (Beckman Coulter). Libraries were quantified using a KAPA Library Quantification Kit Illumina (Kapa Biosystems) and clustered in the Illumina Cbot according to the manufacturer's instructions (Illumina, San Diego, CA, USA). Sequencing was performed using an Illumina HiSeq instrument with a modified protocol for 150 base paired‐end reads.^44^ Read 1 was sequenced with the Illumina read 1 primer. Read 2 was sequenced with a mix of 13 *TRBJ* gene‐specific primers. The minimum coverage for each sampled population was approximately 10 reads per input cell. TCRβ annotation was performed by combining a custom Java script with the BLAST1 program (National Center for Biotechnology Information, National Institutes of Health). Briefly, the *V* and *J* genes were identified first, and the CDR3β was determined by finding the conserved cysteine at the 5′ end and the conserved phenylalanine at 3′ end. Unique TCRβ combinations (V‐D‐J) were collapsed to determine the count.

### Statistical analysis

Significance testing was implemented using JMP software version 14 (SAS Institute Inc., Cary, NC, USA).

## Conflict of Interest

The authors declare no conflicts of interest.
