# Supplementary material for: The peripheral differentiation of human natural killer T cells
Source: Immunol Cell Biol. 2019 Apr 8;97(6):586–96. doi: 10.1111/imcb.12248 (PMC6767057; doi:10.1111/imcb.12248)
Supplement: Supplementary file 1 [file IMCB-97-586-s001.pdf]

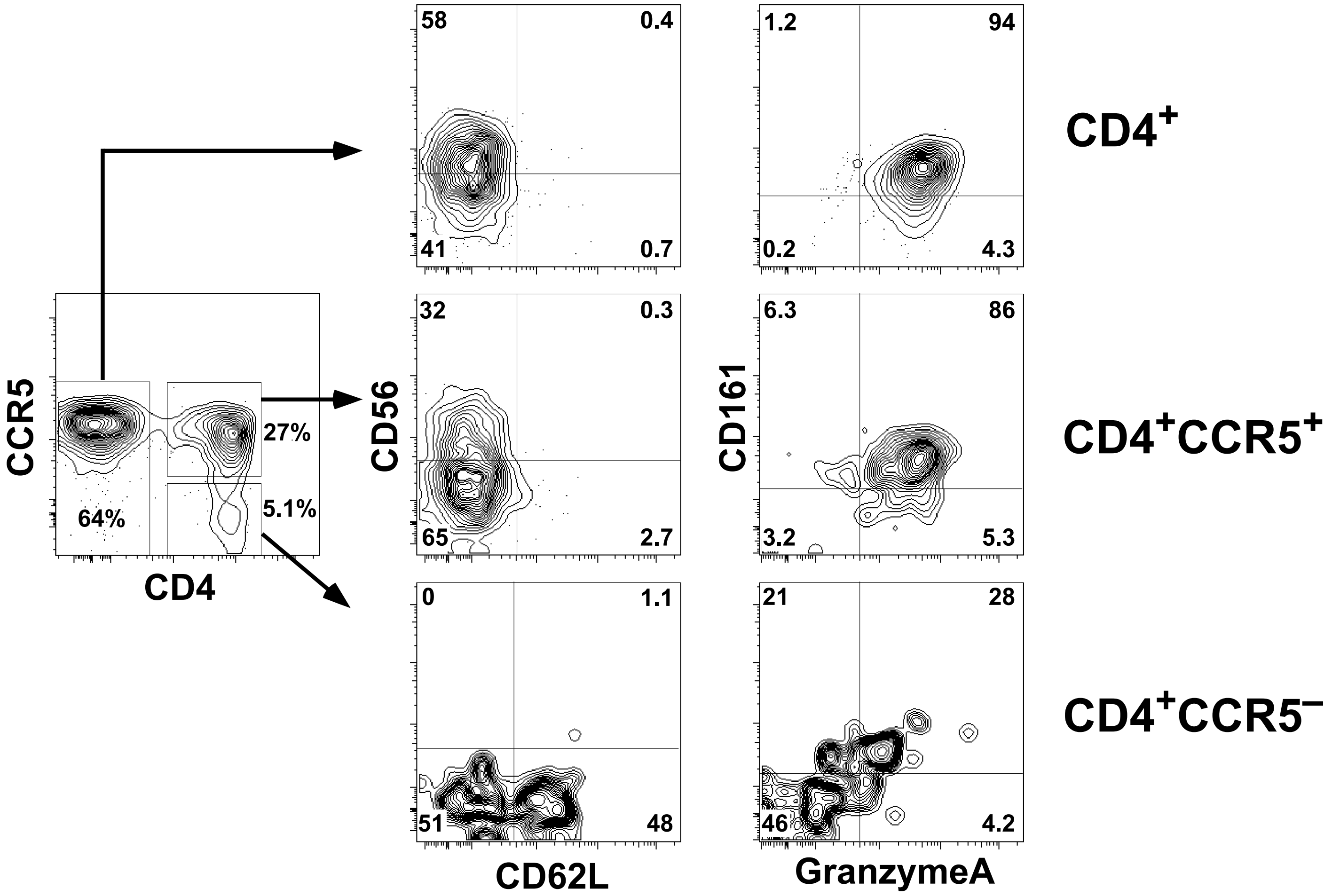

Supplemental Figure 1. Naïve-like phenotype of CD4$^+$CCR5$^–$ NKT cells. Expression of the indicated phenotypic markers is shown for CD4$^+$, CD4$^+$CCR5$^+$, and CD4$^+$CCR5$^–$ NKT cells (PBS57-hCD1d$^+$) from a representative individual. The percentages are averaged across three independent experiments (n = 5 subjects).
