# Supplementary material for: The peripheral differentiation of human natural killer T cells
Source: Immunol Cell Biol. 2019 Apr 8;97(6):586–96. doi: 10.1111/imcb.12248 (PMC6767057; doi:10.1111/imcb.12248)
Supplement: Supplementary file 2 [file IMCB-97-586-s002.pdf]

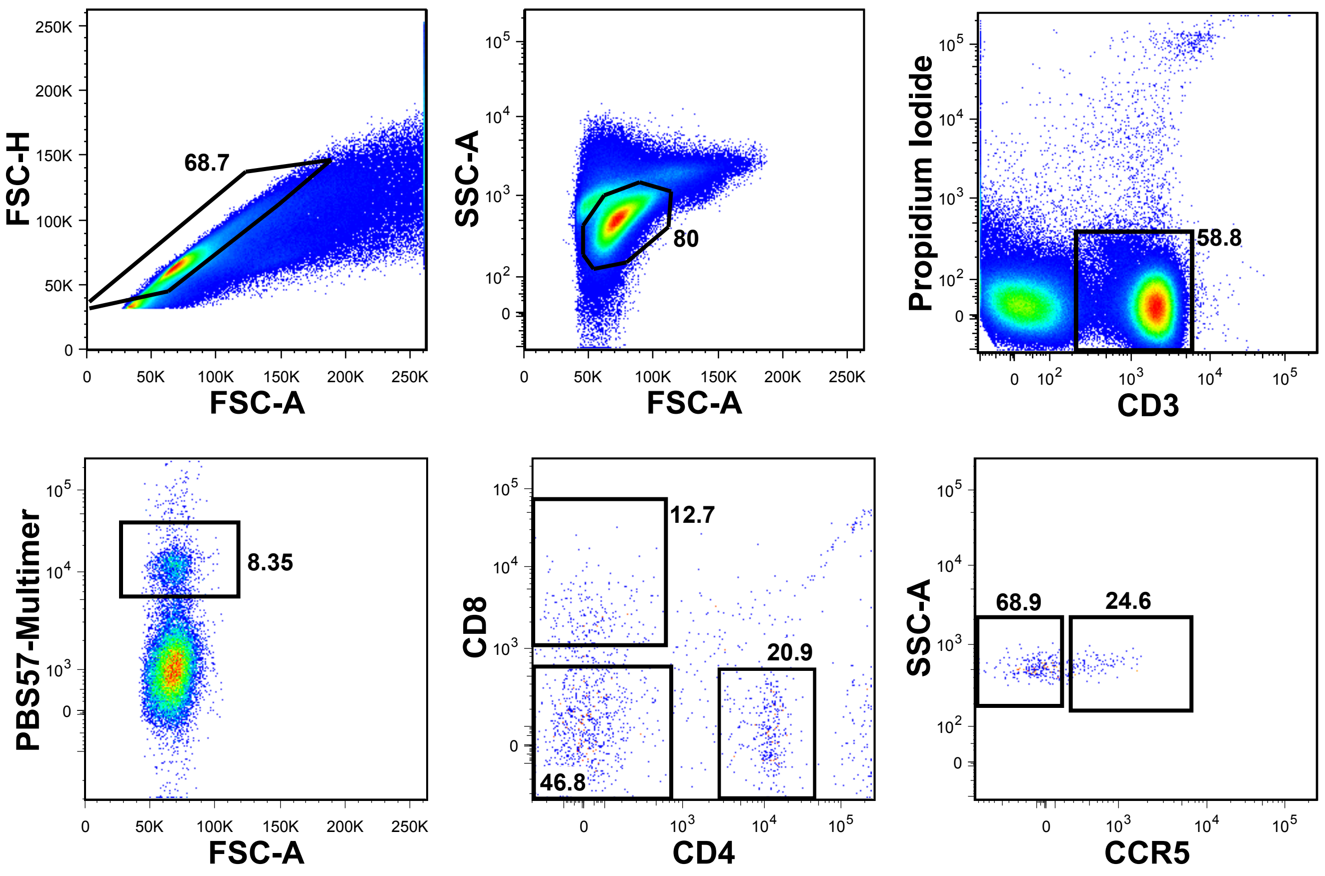

Supplemental Figure 2. Flow cytometric sorting of NKT cell subsets from healthy subjects. Gates were placed serially on singlets, small lymphocytes, and viable $CD3^+$ cells (top panels). NKT cells were identified using the PBS57-hCD1d multimer (bottom left). The $CD4^+$, DN, and $CD8^+$ subsets (bottom middle) were sorted for conventional analysis of the TCR repertoire (Figure 4). The $CD4^+$ subset was further subdivided into $CCR5^-$ and $CCR5^+$ cells (bottom right) for high-throughput analysis of the TCR repertoire (Figure 5).
